# Case Report: Giant cell reparative granuloma of the humerus and femur—two rare cases with atypical skeletal involvement

**DOI:** 10.3389/fmed.2026.1878294

**Published:** 2026-07-07

**Authors:** Fangfang Xu, Qian Li, Fei Dong, Chao Wang

**Affiliations:** Department of Radiology, The Second Affiliated Hospital, Zhejiang University School of Medicine, Hangzhou, China

**Keywords:** case report, computed tomography, giant cell reparative granuloma, histopathological examination, magnetic resonance imaging

## Abstract

**Introduction:**

Giant cell reparative granuloma (GCRG) is a rare benign lesion that typically arises in the mandible and maxilla, whereas involvement of the long bones is extremely uncommon. It is generally considered a reactive, non-neoplastic process associated with prior trauma or surgical intervention.

**Case report:**

We report two rare cases of GCRG involving the extremities: one in the proximal humerus and the other in the distal femur. Both patients had a history of prior mechanical strain or localized trauma. Imaging revealed well-defined expansile osteolytic lesions with heterogeneous internal features and contrast enhancement, initially mimicked giant cell tumor of bone. Surgical intervention was performed in both cases, and the diagnosis of GCRG was confirmed by postoperative histopathological examination.

**Conclusion:**

These cases underscore the importance of including GCRG in the differential diagnosis of osteolytic lesions at atypical skeletal sites, particularly in patients with a history of trauma. Accurate diagnosis relies on the integration of radiologic and histopathological findings to prevent misdiagnosis and guide appropriate management.

## Introduction

Giant cell reparative granuloma (GCRG) is a rare, benign reactive lesion that predominantly arises in the mandible and maxilla (1). It is generally regarded as a reactive proliferative response to surgical trauma or tissue injury and is histologically characterized by multinucleated giant cells, and chronic inflammation (2). Extragnathic involvement is exceedingly uncommon but has been reported in sites such as the temporal bone and, in rare cases, the parietal bone or intracranial structures (3). In addition, Involvement of the extremities is even rarer, particularly in patients with a history of trauma or prior surgery, posing additional diagnostic challenges in clinical practice.

Here, we report two cases of GCRG involving the extremities, one in the proximal humerus and the other in the distal lower limb, both associated with prior mechanical strain or trauma at the affected site. Although initial imaging findings suggested bone tumors, postoperative histopathological examination confirmed the diagnosis of GCRG, providing additional insight into this rare entity.

### Case report 1

A 45-year-old woman presented with an 8-month history of left shoulder pain and weakness following heavy lifting, with progressive worsening over the preceding two months and movement-related discomfort. Imaging studies from the referring institution, including X-ray and computed tomography (CT), were unavailable. Her medical history was notable for well-controlled hypertension of six years’ duration. Physical examination revealed no significant abnormalities.

**Figure 1:**
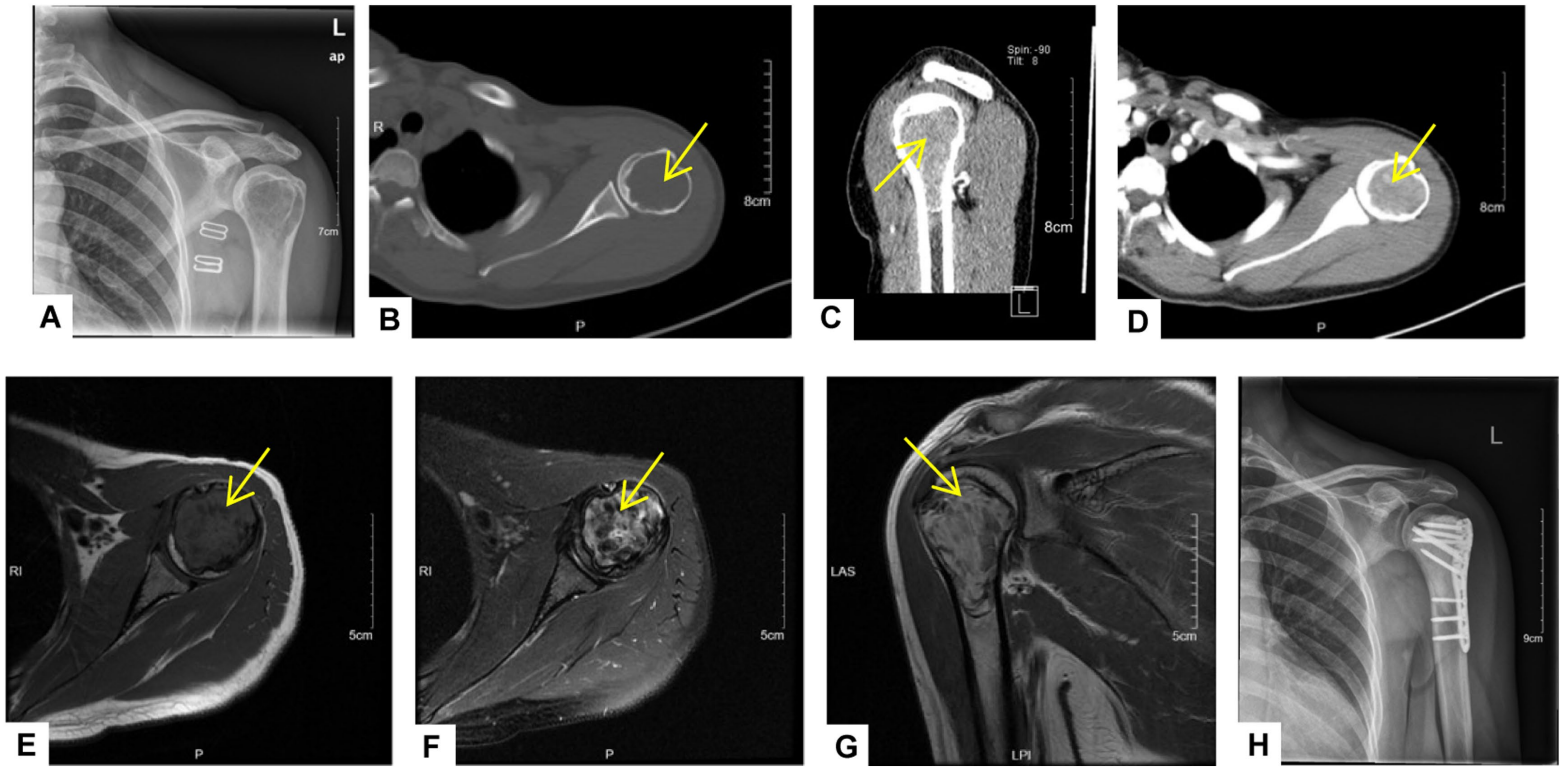
Radiographic, CT, MRI, and postoperative findings of the proximal humeral lesion. **(A)** Plain radiography showed a well-defined osteolytic lesion in the left humeral head, with a multiloculated honeycomb-like appearance and fine internal osseous septations, suggesting a multiloculated intraosseous process. **(B)** Axial CT bone-window image demonstrated an expansile intraosseous lesion with a sclerotic margin and cortical thinning, without evident cortical breakthrough, periosteal reaction, or extraosseous soft-tissue mass, further delineating the lesion extent and internal architecture (arrow). **(C,D)** Contrast-enhanced CT further delineated the extent and internal architecture of the proximal humeral lesion, demonstrating marked heterogeneous enhancement and a well-defined lesion boundary (arrow). **(E)** Axial T1WI MRI demonstrated predominantly low-to-intermediate signal intensity within the lesion (arrow). **(F)** Axial T2WI demonstrated heterogeneous mixed hyperintense and hypointense signals, reflecting variable internal tissue components (arrow). **(G)** On coronal contrast-enhanced T1WI, the lesion demonstrated marked enhancement with focal non-enhancing low-signal areas, suggestive of hemosiderin deposition, hemorrhagic components, or residual osseous trabeculae (arrow). **(H)** Postoperative radiography showed bone cementation and internal fixation of the proximal left humerus, with preserved shoulder alignment and no evident hardware-related complications. Original radiographic, CT, MRI, and postoperative images were obtained from the Second Affiliated Hospital, Zhejiang University School of Medicine.

Initial radiographs of the proximal left humerus demonstrated a well-defined osteolytic lesion in the left humeral head, with a multiloculated honeycomb-like appearance and fine internal osseous septations (Figure 1A). Axial CT bone-window imaging further demonstrated an expansile intraosseous lesion with a sclerotic margin and cortical thinning, without evident cortical breakthrough, periosteal reaction, or extraosseous soft-tissue mass (Figure 1B). Contrast-enhanced CT delineated the extent and internal architecture of the proximal humeral lesion, which measured approximately 39 × 29 × 57 mm and demonstrated marked heterogeneous enhancement, with an attenuation value of approximately 87 Hounsfield units (HU) (Figures 1C,D). Magnetic resonance imaging (MRI) of the left shoulder showed iso- to hypointense signals on axial T1-weighted imaging (T1WI) (Figure 1E) and heterogeneous hyperintense and hypointense signals on axial T2-weighted imaging (T2WI) (Figure 1F). On coronal contrast-enhanced T1WI, the lesion demonstrated marked enhancement with focal non-enhancing low-signal areas, suggestive of hemosiderin deposition, hemorrhagic components, or residual osseous trabeculae (Figure 1G). Based on these imaging findings, a bone neoplasm, including a giant cell tumor of bone, was considered.

After contraindications were excluded, the patient underwent intralesional curettage and inactivation of the proximal humeral lesion, followed by bone cementation and internal fixation. The procedure was uneventful. Postoperative histopathological examination revealed abundant spindle cells with numerous multinucleated giant cells and mononuclear cells, accompanied by hemorrhage, secondary fibroblast proliferation, and hyalinization, consistent with an osteoclast-like giant cell-rich lesion (Supplementary Figure S1) and supporting the diagnosis of GCRG. Postoperative radiograph showed satisfactory reconstruction of the proximal left humerus, with preserved shoulder alignment and no evident hardware-related complications (Figure 1H). The patient had an uneventful postoperative recovery and underwent annual follow-up imaging, with no evidence of recurrence.

**Figure 2:**
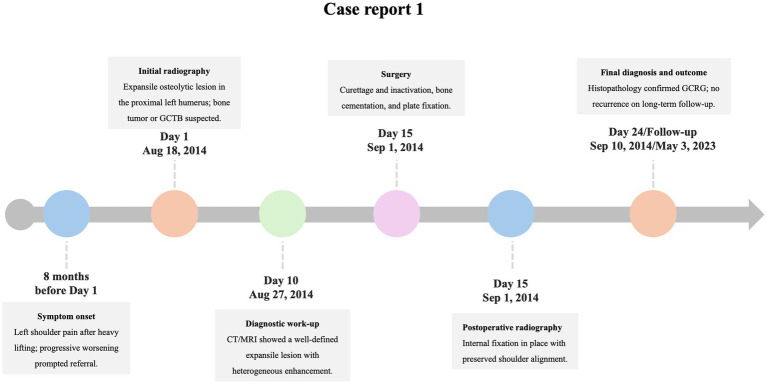
Timeline of the clinical course in Case report 1. The timeline summarizes the sequential clinical course of Case report 1, beginning with shoulder pain after mechanical strain and progressing through imaging detection of the proximal humeral lesion, surgical curettage with local inactivation, bone cementation and internal fixation, histopathological confirmation of GCRG, and no recurrence was noted during follow-up.

Overall, the clinical course was characterized by initial shoulder pain, radiographic detection of an expansile osteolytic lesion, surgical management, histopathological confirmation of GCRG, and absence of recurrence during annual follow-up (Figure 2).

### Case report 2

A 26-year-old man presented with intermittent mild stabbing pain in the right knee after a fall 10 days earlier, which was exacerbated by physical activity. Radiography performed at a local hospital suggested a possible giant cell tumor of the distal right femur; however, the original report was unavailable. He had no significant medical history, and physical examination was unremarkable.

**Figure 3:**
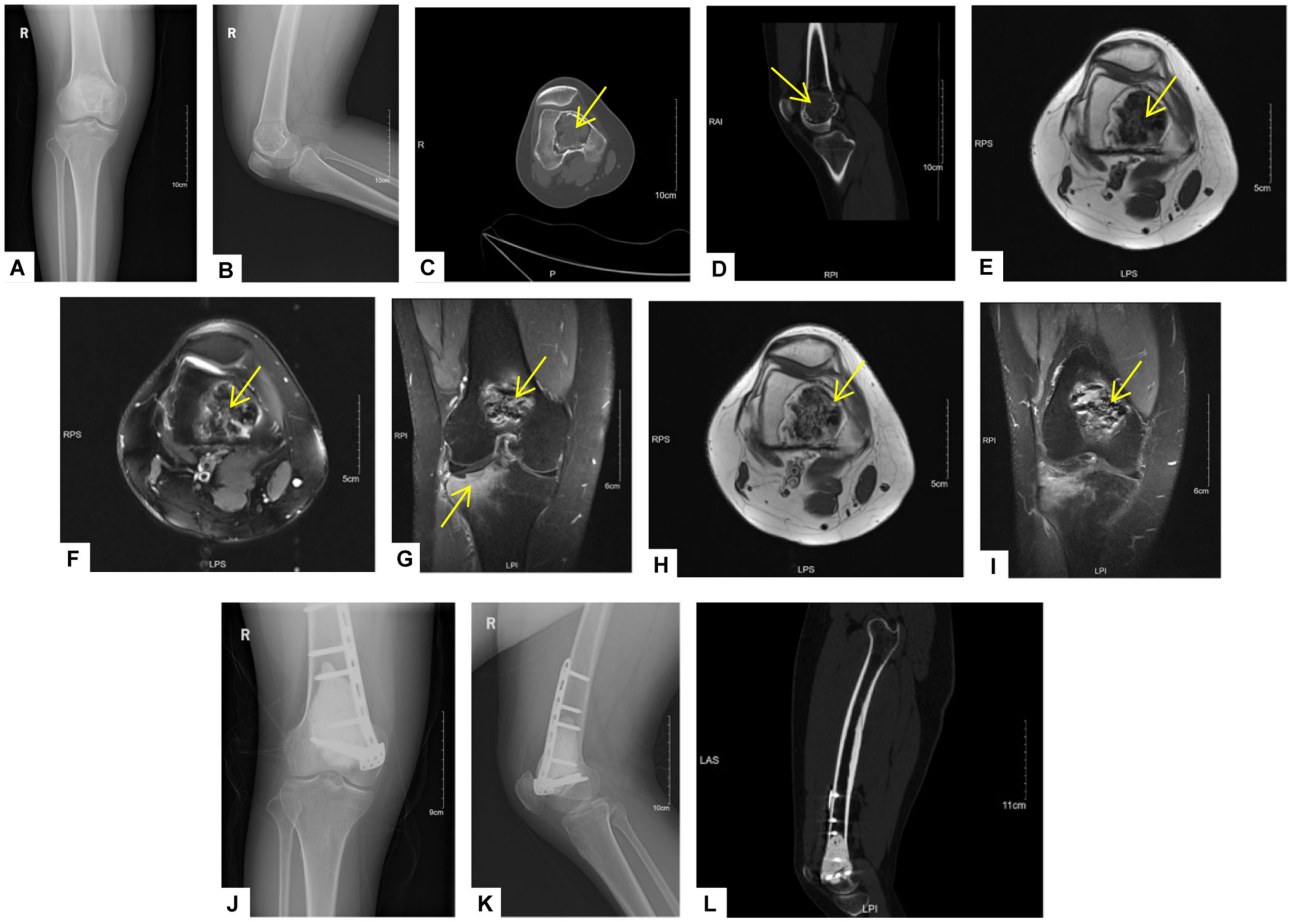
Radiographic, CT, MRI, postoperative, and follow-up findings of the distal femoral lesion. **(A, B)** Anteroposterior and lateral radiographs of the right knee revealed a well-defined osteolytic lesion located in the distal femur. The lesion demonstrated an expansile growth pattern with internal reticulated osseous septa. **(C)** Contrast-enhanced CT demonstrated a lobulated lesion with heterogeneous internal density and focal ossified components, further delineating its internal architecture. A thin sclerotic rim was observed along the lesion margin, suggesting a well-defined boundary (arrow). **(D)** Mild post-contrast enhancement was observed, suggesting enhancing soft-tissue components within the lesion (arrow). **(E)** Axial T1WI showed mixed iso- and hypointense signals (arrow). **(F)** Axial T2WI demonstrated heterogeneous signal intensity, consistent with the complex internal architecture of the lesion (arrow). **(G)** Coronal contrast-enhanced T1WI demonstrated bone marrow edema and a focal fracture within the tibial plateau, consistent with the recent traumatic episode (arrow). **(H,I)** Contrast-enhanced MRI demonstrated marked enhancement of most of the lesion, whereas focal low-signal areas remained non-enhancing, compatible with hemosiderin deposition or residual hemorrhagic components (arrow). **(J)** Postoperative radiograph confirmed stable internal fixation of the distal femur after surgical treatment. **(K,L)** Follow-up radiograph and CT demonstrated stable postoperative changes without evidence of local recurrence. Original radiographic, CT, MRI, postoperative, and follow-up images were obtained from the Second Affiliated Hospital, Zhejiang University School of Medicine.

Initial anteroposterior and lateral radiographs of the right knee revealed a well-defined osteolytic lesion in the distal right femur, with expansile remodeling and internal reticulated osseous septa (Figures 3A,B). Contrast-enhanced CT of the right knee demonstrated an expansile osteolytic lesion in the distal femur, measuring approximately 41 × 40 × 43.9 mm. The lesion exhibited heterogeneous internal density, a lobulated contour, peripheral enhancement, and areas of visible ossification. A thin low-attenuation rim was observed along the lesion margin, suggesting a well-defined boundary (Figure 3C). Furthermore, mild post-contrast enhancement was observed of the lesion (Figure 3D). Contrast-enhanced MRI of the right knee showed a well-defined, mildly lobulated expansile mass arising from the distal femur. The lesion displayed mixed iso- to hypointense signals on T1WI (Figure 3E), while T2WI showed heterogeneous hyper- to hypointense signals (Figure 3F). Additionally, bone marrow edema and a focal fracture were also noted in the tibial plateau (Figure 3G). Post-contrast images demonstrated marked lesion enhancement, whereas focal low-signal areas on T1WI and T2WI remained non-enhancing, suggestive of hemosiderin deposition (Figures 3H,I). Based on these imaging findings, a preliminary diagnosis of giant cell tumor of bone was considered.

After exclusion contraindications, the patient underwent resection of the right femoral lesion, internal fixation, and autologous bone grafting from the right ilium. The procedure was uneventfully. Postoperative pathological examination revealed a heterogeneous lesion with areas of hemorrhage (Supplementary Figure S2A). Histologically, the lesion comprised multiple small foci of proliferating short spindle cells, multinucleated giant cells, abundant hemosiderin deposition, and foamy histiocytes. The spindle cells showed prominent collagen deposition, myxoid degeneration, and focal ossification (Supplementary Figure S2B). Overall, the histomorphological findings supported the diagnosis of GCRG. Postoperative radiographs demonstrated stable internal fixation in the distal right femur without complications (Figure 3J). The patient experienced an uneventful recovery. Regular follow-up imaging was performed, and the most recent radiographs and CT scans revealed no evidence of recurrence (Figures 3K,L).

**Figure 4:**
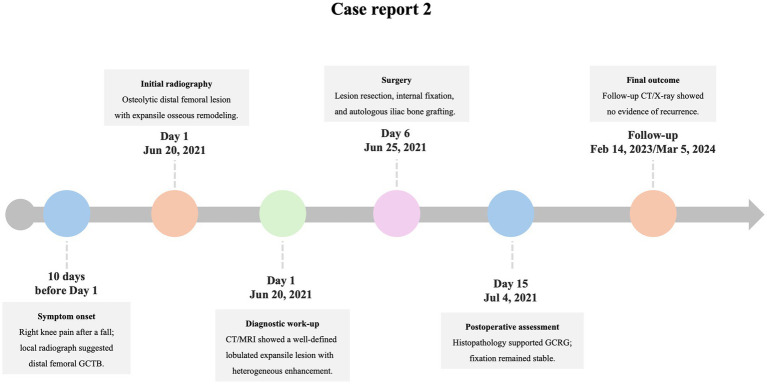
Timeline of the clinical course in Case report 2. The timeline outlines the sequential clinical course of Case report 2, from post-traumatic knee pain to radiographic, CT, and MRI evaluation of the distal femoral lesion. Surgical resection, internal fixation, and autologous bone grafting were subsequently performed, followed by histopathological confirmation of GCRG. Follow-up imaging showed no evidence of recurrence.

In brief, the diagnostic and therapeutic course of this case proceeded from post-traumatic knee pain to imaging-based evaluation, surgical management, histopathological confirmation of GCRG, and follow-up assessment (Figure 4).

## Discussion

GCRG is a rare benign, non-neoplastic intraosseous reactive lesion that may be associated with local trauma, dysregulated repair, developmental disturbance, or inflammation. Histologically, it is characterized by fibrocellular stroma, hemorrhagic foci, osteoclast-like multinucleated giant cells, and occasional woven bone or osteoid formation (2). GCRG was first described by Jaffe in 1953 and accounts for approximately 7% of benign jaw lesions, with the majority of cases occurring in individuals under 30 years of age (4, 5). Although the jawbone is the most common site, GCRG may occasionally arise in other skeletal locations, which presents a significant diagnostic challenge. Here, we report two rare cases of GCRG involving the humerus and femur. Notably, both patients had a history of localized trauma or prior mechanical strain. The final diagnosis of GCRG was established based on comprehensive imaging findings in combination with postoperative histopathological examination. These cases provide valuable insights into the diagnosis of rare extragnathic GCRG and highlight the importance of considering prior trauma during the diagnostic evaluation.

The pathogenesis of GCRG is thought to involve intraosseous hemorrhage or vascular injury within a fibrovascular matrix, which stimulates mononuclear stromal cells and promotes cytokine-mediated recruitment and fusion of monocytes into osteoclast-like multinucleated giant cells (1, 2). Although GCRG most commonly occurs in the jawbone, it can also develop at extragnathic sites after surgical trauma or bone injury, supporting its classification as a reparative process rather than a true neoplasm.

Radiologically, GCRG typically presents as an expansile osteolytic bone lesion. Radiographs and CT imaging often reveal a multiloculated “soap-bubble” appearance, accompanied by cortical thinning, mild osseous expansion, and no aggressive periosteal reaction (6). On contrast-enhanced CT and MRI, GCRG commonly demonstrates heterogeneous enhancement, internal septations, and mixed signal intensity, reflecting hemorrhage or hemosiderin deposition. These components may appear as low-signal foci on both T1WI and T2WI (7). Notably, a thin sclerotic rim may also be present, indicating a reactive bone response (8). In contrast, giant cell tumors of bone (GCTB) typically present as an eccentric lytic lesion adjacent to the articular surface, with more pronounced cortical destruction and fewer hemosiderin-related low-signal areas (8). Aneurysmal bone cysts (ABCs) are characterized by fluid–fluid levels on MRI, reflecting layered blood products, but typically lack prominent hemosiderin deposition (9). In our humeral and femoral cases, imaging showed well-demarcated expansile hypodense lesions with heterogeneous contrast enhancement, cortical thinning without periosteal reaction, and internal foci of non-enhancing low signal intensity on both T1- and T2-weighted sequences, consistent with hemosiderin deposition. These findings align with the established radiologic features of GCRG and may help distinguish it from more aggressive entities, such as GCTB or ABCs.

Pathologically, GCRG is characterized by spindle-shaped fibroblastic stromal cells, multinucleated giant cells that often cluster around hemorrhagic areas, focal osteoid or reactive bone formation, and variable hemosiderin deposition (2, 10). These findings were consistent with the pathological features in our cases. ABCs consist of blood-filled cystic cavities separated by fibrous septa containing fibroblasts, multinucleated giant cells, and woven bone, but typically lack hemosiderin deposition (11). GCTB usually shows a more uniform distribution of giant cells, increased mitotic activity, and frequent H3F3A mutations (12). In both the humeral and femoral lesions, histological examination revealed stromal spindle cells, numerous multinucleated giant cells, hemorrhagic foci, reactive ossification, low mitotic activity, and focal hemosiderin deposition, supporting the diagnosis of GCRG and distinguishing it from GCTB and ABCs.

Surgical curettage or resection remains the primary treatment modality for GCRG, with en bloc resection recommended for larger or locally aggressive lesions. Following lesion excision, cortical preservation and bone grafting may be required to restore structural stability, particularly in long bones. Reported postoperative recurrence rates range from 2 to 25%, most often after simple curettage, although recurrent lesions are usually manageable with repeat curettage (13–15). In our humeral and femoral cases, surgical treatment, lesion inactivation, and internal fixation were successfully performed, with no recurrence observed in either patient during annual follow-up.

In conclusion, we report two rare cases of GCRG involving the extremity bones, in which comprehensive imaging evaluation combined with histopathological examination established the final diagnosis. These cases broaden the clinical understanding of atypical extragnathic GCRG and highlight the diagnostic value of integrating radiological and pathological findings to reduce misdiagnosis and avoid unnecessary overtreatment.

## Data Availability

The raw data supporting the conclusions of this article will be made available by the authors, without undue reservation.

## Supplementary material

SUPPLEMENTARY FIGURE S1 Histopathological findings of the proximal humeral lesion. Microscopic examination revealed spindle cell proliferation with numerous multinucleated giant cells and mononuclear cells, accompanied by hemorrhage, fibroblastic proliferation, and hyalinization. Original histopathological image was obtained from the Second Affiliated Hospital, Zhejiang University School of Medicine.

SUPPLEMENTARY FIGURE S2 Histopathological findings of the distal femoral lesion. **(A)** The lesion showed hemorrhagic change. **(B)** Histological examination demonstrated short spindle cells, multinucleated giant cells, hemosiderin-laden areas, and foam cells, with associated collagen deposition, myxoid change, and focal ossification. Original gross pathological and histopathological images were obtained from the Second Affiliated Hospital, Zhejiang University School of Medicine.
