# Supplementary figures and images for: Case Report: Giant cell reparative granuloma of the humerus and femur—two rare cases with atypical skeletal involvement
Source: Front Med (Lausanne). 2026 Jul 7;13:1878294. doi: 10.3389/fmed.2026.1878294 (PMC13384876; doi:10.3389/fmed.2026.1878294)

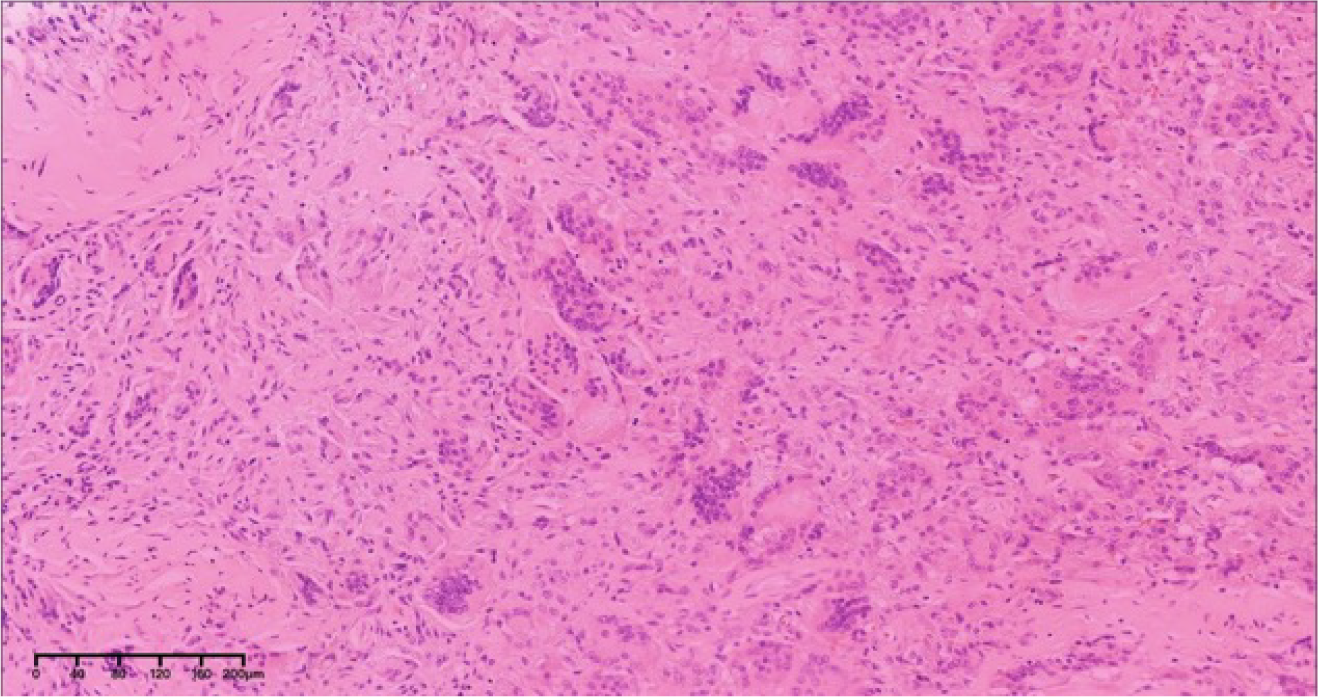

Supplement: SUPPLEMENTARY FIGURE S1 — Histopathological findings of the proximal humeral lesion. Microscopic examination revealed spindle cell proliferation with numerous multinucleated giant cells and mononuclear cells, accompanied by hemorrhage, fibroblastic proliferation, and hyalinization. Original histopathological image was obtained from the Second Affiliated Hospital, Zhejiang University School of Medicine. [file Image_1.tif]

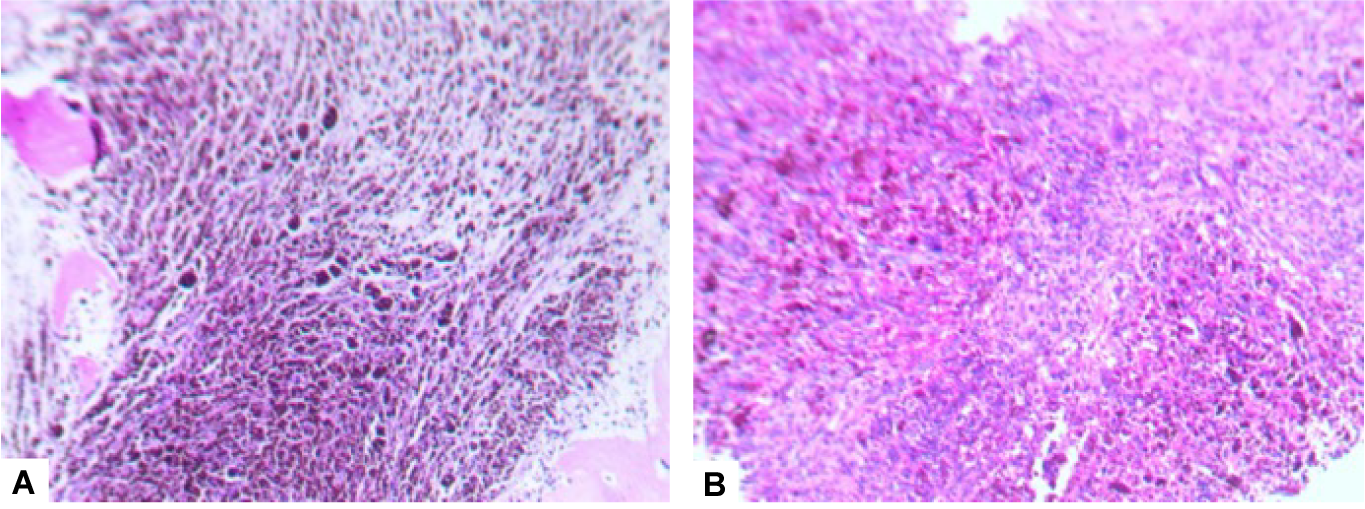

Supplement: SUPPLEMENTARY FIGURE S2 — Histopathological findings of the distal femoral lesion. (A) The lesion showed hemorrhagic change. (B) Histological examination demonstrated short spindle cells, multinucleated giant cells, hemosiderin-laden areas, and foam cells, with associated collagen deposition, myxoid change, and focal ossification. Original gross pathological and histopathological images were obtained from the Second Affiliated Hospital, Zhejiang University School of Medicine. [file Image_2.tif]
